# Novel Bioactivity of Ellagic Acid in Inhibiting Human Platelet Activation

**DOI:** 10.1155/2013/595128

**Published:** 2013-02-21

**Authors:** Yi Chang, Wei-Fan Chen, Kuan-Hung Lin, Cheng-Ying Hsieh, Duen-Suey Chou, Li-Jyun Lin, Joen-Rong Sheu, Chao-Chien Chang

**Affiliations:** ^1^Department of Anesthesiology, Shin Kong Wu Ho-Su Memorial Hospital, Taipei 111, Taiwan; ^2^School of Medicine, Fu Jen Catholic University, Taipei 242, Taiwan; ^3^Graduate Institute of Medical Sciences, Taipei Medical University, 250 Wu-Hsing Street, Taipei 110, Taiwan; ^4^Department of Pharmacology, Taipei Medical University, 250 Wu-Hsing Street, Taipei 110, Taiwan; ^5^Department of Cardiology, Cathay General Hospital, Section 4, No. 280, Jen-Ai Road, Taipei 106, Taiwan

## Abstract

Pomegranates are widely consumed either as fresh fruit or in beverage form as juice and wine. Ellagic acid possesses potent antioxidative properties; it is known to be an effective phytotherapeutic agent with antimutagenic and anticarcinogenic qualities. Ellagic acid (20 to 80 **μ**M) exhibited a potent activity in inhibiting platelet aggregation stimulated by collagen; however, it did not inhibit platelet aggregation stimulated by thrombin, arachidonic acid, or U46619. Treatment with ellagic acid (50 and 80 **μ**M) significantly inhibited platelet activation stimulated by collagen; this alteration was accompanied by the inhibition of relative [Ca^2+^]_*i*_ mobilization, and the phosphorylation of phospholipase C (PLC)**γ**2, protein kinase C (PKC), mitogen-activated protein kinases (MAPKs), and Akt, as well as hydroxyl radical (OH^●^) formation. In addition, ellagic acid also inhibited p38 MAPK and Akt phosphorylation stimulated by hydrogen peroxide. By contrast, ellagic acid did not significantly affect PKC activation and platelet aggregation stimulated by PDBu. This study is the first to show that, in addition to being considered a possible agent for preventing tumor growth, ellagic acid possesses potent antiplatelet properties. It appears to initially inhibit the PLC*γ*2-PKC cascade and/or hydroxyl radical formation, followed by decreased phosphorylation of MAPKs and Akt, ultimately inhibiting platelet aggregation.

## 1. Introduction

**Figure 1:**
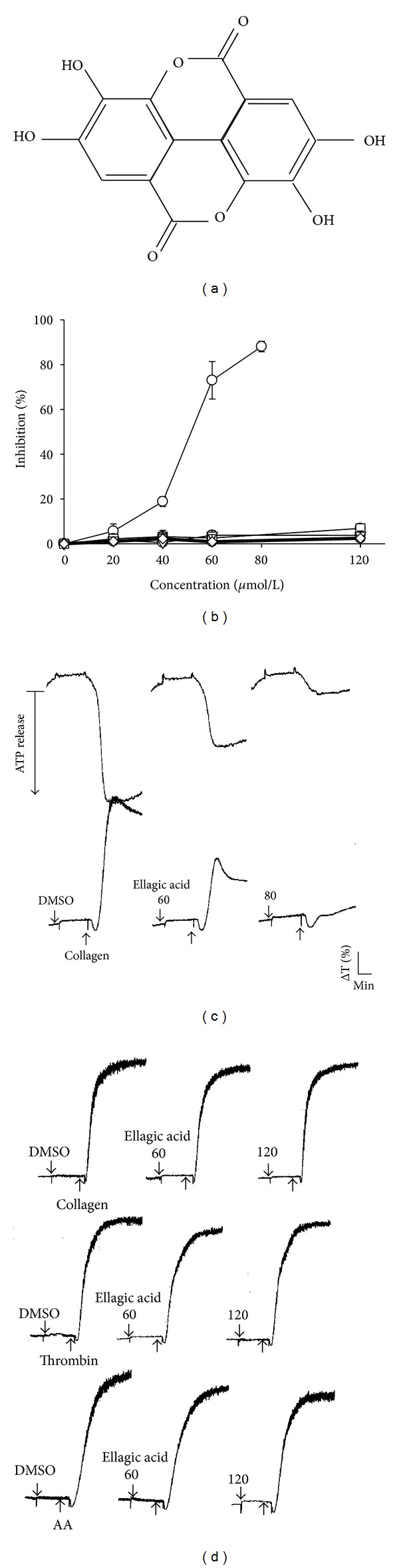
Inhibitory effects of ellagic acid on platelet aggregation in washed human platelets. (a) Chemical structure of ellagic acid. (b)–(d) Washed platelets (3.6 × 10^8^ cells/mL) were preincubated with 20–120 *μ*M ellagic acid or 0.5% DMSO; this was followed by the addition of 1 *μ*g/mL collagen (○), 1 *μ*M U46619 (*▽*), 60 *μ*M arachidonic acid (□), or 0.05 IU/mL thrombin (*⋄*) to trigger platelet aggregation and an ATP-release reaction ((c), upper tracings). The data in (b) are presented as the means ± S.E.M. (*n* = 4). The profiles ((c) and (d)) are representative examples of 4 similar experiments.

Epidemiological studies have shown that an inverse relationship exists between diets rich in fruits, vegetables, and spices and the risk of all causes of death from cancer and cardiovascular diseases (CVD) [1]. Fresh and processed fruits and food products contain high levels of a diverse range of phytochemicals of which polyphenols including hydrolyzable tannins (ellagitannins (ETs) and gallotannins) and condensed tannins (proanthocyanidins), anthocyanins, and other flavonoids make up a large proportion [2]. Pomegranates (*Punica granatum* L.) are widely consumed both as fresh fruit and in beverage form as juice or wine [3]. Commercial pomegranate juice contains high levels of polyphenols, including ellagic acid in its free (Figure 1(a)) and bound forms (such as ETs and ellagic acid glycosides), gallotannins, anthocyanins (cyanidin, delphinidin, and pelargonidin glycosides), and other flavonoids [2, 3]. The most abundant of these polyphenols is punicalagin, an ET implicated as the bioactive constituent responsible for over 50% of the juice's potent antioxidant activity [3].

Ellagic acid displays antioxidant properties such as the ability to scavenge free radicals and chelate metal ions [4, 5]. Ellagic acid is one of the most interesting compounds among the numerous natural substances that possess proapoptotic qualities, and which have been investigated in vitro and in vivo [6]. Follow-up studies have shown that ellagic acid is an effective phytotherapeutic agent that exerts antimutagenic and anticarcinogenic effects through diverse cellular mechanisms. These include the induction of cell cycle arrest and apoptosis and the prevention of carcinogens binding to DNA; these actions might inhibit the onset of cancer or the proliferation of tumors [6–9].

Intravascular thrombosis is one of the generators of various CVDs. Initiation of an intraluminal thrombosis is believed to involve platelet adherence and aggregation. Platelet activation and aggregation are common denominators in atherothrombotic events, and platelet aggregation might play a crucial role in the atherothrombotic process [10]. Therefore, investigation of antiplatelet agents to inhibit atherothrombotic events (myocardial infarction, ischemic stroke, and vascular death) is warranted. 

Even though studies have reported that an extract of armagnac (rich in ellagic acid and ETs) significantly inhibits platelet aggregation in human platelets [11, 12], very few data had been published so far on the effect of ellagic acid in human platelets. One study reported that ellagic acid significantly stimulated platelet activation in rabbit platelets [13]. From our preliminary finding it is shown that ellagic acid (60 *μ*M) markedly inhibited platelet aggregation stimulated by collagen in washed human platelets. This discrepancy might result from species-specific characteristics of platelets. We thus systematically examined the influence of ellagic acid in human platelets and further characterized the mechanisms of ellagic acid-mediated inhibition of platelet activation. 

## 2. Materials and Methods

### 2.1. Materials

Ellagic acid, collagen (type I), luciferin-luciferase, arachidonic acid (AA), phorbol-12,13-dibutyrate (PDBu), 5,5-dimethyl-1 pyrroline N-oxide (DMPO), U46619, and thrombin were purchased from Sigma (St. Louis, MO, USA). Fura 2-AM was obtained from Molecular Probe (Eugene, OR). The antiphospho-p38 mitogen-activated protein kinase (MAPK) Ser^182^ monoclonal antibody (mAb) was obtained from Santa Cruz (Santa Cruz, CA, USA). The anti-p38 MAPK and antiphospho-c-Jun N-terminal kinase (JNK) (Thr^183^/Tyr^185^) mAbs, antiphospholipase C*γ*2 (PLC*γ*2), antiphospho (Tyr^759^) PLC*γ*2, antiphospho-(Ser) PKC substrate, and antiphospho-p44/p42 extracellular signal-regulated kinase (ERK) (Thr^202^/Tyr^204^) polyclonal antibodies (pAbs) were purchased from Cell Signaling (Beverly, MA, USA). Antiphospho-Akt (Ser^473^) and anti-Akt mAbs were obtained from Biovision (Mountain View, CA, USA). The anti-*α*-tubulin mAb was obtained from NeoMarkers (Fremont, CA, USA). The Hybond-P polyvinylidene difluoride (PVDF) membrane, enhanced chemiluminescence (ECL) Western blotting detection reagent and analysis system, horseradish-peroxidase- (HRP-) conjugated donkey antirabbit immunoglobulin G (IgG), and sheep antimouse IgG were obtained from Amersham (Buckinghamshire, UK). The ellagic acid was dissolved in 0.5% dimethyl sulfoxide (DMSO) and stored at 4°C until use.

### 2.2. Platelet Aggregation

The study was approved by the Institutional Review Board of Taipei Medical University and conformed to the principles outlined in the Helsinki Declaration; all human volunteers provided informed consent to participate. 

Human platelet suspensions were prepared as previously described [10]. In brief, blood was collected from healthy human volunteers who had taken no medication during the preceding 2 weeks and was mixed with an acid-citrate-dextrose (ACD) solution (9 : 1, v/v). After centrifugation, the supernatant (platelet-rich plasma; PRP) was supplemented with 0.5 *μ*M prostaglandin E_1_ (PGE_1_) and 6.4 IU/mL heparin. Washed platelets were suspended in a Tyrode's solution containing 3.5 mg/mL bovine serum albumin (BSA). The final concentration of Ca^2+^ in the Tyrode's solution was 1 mmol/L.

A turbidimetric method was used to measure platelet aggregation [10], with a Lumi-Aggregometer (Payton, Scarborough, ON, Canada). Platelet suspensions (3.6 × 10^8^ cells/mL) were preincubated with various concentrations of ellagic acid (20–120 *μ*M) or an isovolumetric solvent control (0.5% DMSO) for 3 min; thereafter, agonists (i.e., collagen, thrombin, U46619, and AA) were added. The reaction was allowed to proceed for 6 min, and the extent of aggregation was expressed as a percentage of the control (absence of ellagic acid) in light-transmission units. When measuring ATP release, 20 *μ*L of a luciferin/luciferase mixture was added 1 min before the addition of agonists, and ATP release was compared to that of the control.

### 2.3. Measurement of Relative [Ca^2+^]_*i*_ Mobilization by Fura 2-AM Fluorescence

Citrated whole blood was centrifuged at 120 ×g for 10 min. The supernatant was incubated with 5 *μ*M Fura 2-AM for 1 h. Human platelets were then prepared as described. Finally, the external Ca^2+^ concentration of the platelet suspensions was adjusted to 1 mM. Relative [Ca2+]_**i**_ mobilization was measured using a fluorescence spectrophotometer (CAF 110, Jasco, Tokyo, Japan) with excitation wavelengths of 340 and 380 nm, and an emission wavelength of 500 nm [10].

### 2.4. Immunoblotting

Washed platelets (1.2 × 10^9^ cells/mL) were preincubated with 50 or 80 *μ*M ellagic acid or a solvent control for 3 min, followed by the addition of agonists to trigger platelet activation. The reaction was stopped, and platelets were immediately resuspended in 200 *μ*L of a lysis buffer. Samples containing 80 *μ*g of protein were separated using a 12% sodium dodecylsulfate polyacrylamide gel electrophoresis (SDS-PAGE); the proteins were electrotransferred by semidry transfer (Bio-Rad, Hercules, CA, USA). The blots were blocked with TBST (10 mM Tris-base, 100 mM NaCl, and 0.01% Tween 20) containing 5% BSA for 1 h and then probed with various primary antibodies. Membranes were incubated with HRP-linked antimouse IgG or antirabbit IgG (diluted 1 : 3000 in TBST) for 1 h. Immunoreactive bands were detected using an ECL system. The bar graph depicts the ratios of semiquantitative results obtained by scanning the reactive bands and quantifying their optical density using videodensitometry (Bio-profil; Biolight Windows Application V2000.01; Vilber Lourmat, France).

### 2.5. Measurement of Hydroxyl Radicals Using Electron Spin Resonance (ESR) Spectrometry

We conducted ESR using a Bruker EMX ESR spectrometer as described previously [14]. In brief, platelet suspensions (3.6 × 10^8^ cells/mL) were preincubated with 50 or 80 *μ*M ellagic acid or a solvent control for 3 min; thereafter, 1 *μ*g/mL collagen was added. The reaction was allowed to proceed for 5 min, followed by the addition of 100 *μ*M DMPO for the ESR study. The rate of free radical scavenging activity was defined by the following equation: inhibition rate = 1 – [signal height (ellagic acid)/signal height (solvent control)] [14].

### 2.6. Data Analysis

Experimental results are expressed as the means ± S.E.M. and are accompanied by the number of observations. The data were assessed using the analysis of variance (ANOVA). If the results showed significant differences among group means, then each group was compared using the Newman-Keuls method. A *P* value of <0.05 was considered statistically significant.

## 3. Results

### 3.1. Effects of Ellagic Acid on Human Platelet Aggregation

In washed human platelets, the addition of ellagic acid (20 to 120 *μ*M) exhibited potent effects in inhibiting platelet aggregation (Figure 1(b)) and the ATP-release reaction (Figure 1(c)) stimulated by treatment with 1 *μ*g/mL collagen. However, ellagic acid did not significantly inhibit platelet aggregation stimulated by any of the following agonists: 0.05 U/mL thrombin, 1 *μ*M U46619 (a prostaglandin endoperoxide), or 60 *μ*M AA (Figure 1(d)); these limited effects were observed even at concentrations of ellagic acid up to 120 *μ*M (Figures 1(b) and 1(d)). The 50% inhibitory concentration (IC_50_) value of ellagic acid for platelet aggregation induced by collagen was approximately 50 *μ*M (Figure 1(b)). The solvent control (0.5% DMSO) did not significantly affect platelet aggregation stimulated by agonists (Figures 1(c) and 1(d)). When platelets were preincubated with ellagic acid at a higher concentration of 120 *μ*M or 0.5% DMSO for 10 min, followed by 2 washes with Tyrode's solution, there were no significant differences between the aggregation curves of either platelet preparation stimulated by collagen (1 *μ*g/mL), indicating that the effect of ellagic acid in inhibiting platelet aggregation occurred in a reversible and noncytotoxic manner (data not shown). In subsequent experiments, we used collagen as an agonist to explore the inhibitory effects and mechanisms of ellagic acid in platelet activation.

### 3.2. Influence of Ellagic Acid in Relative [Ca^2+^]_*i*_ Mobilization and Phospholipase C*γ*2 (PLC*γ*2) and Protein Kinase C (PKC) Activation Stimulated by Collagen

**Figure 2:**
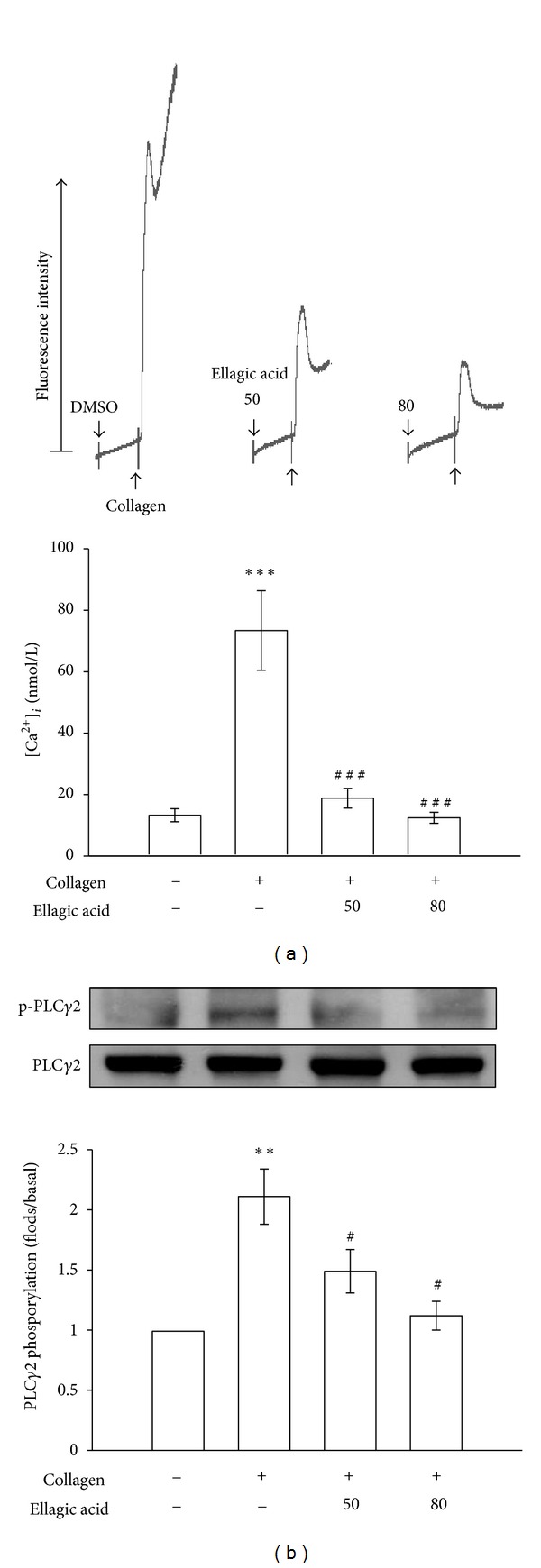
Inhibitory effect of ellagic acid on relative [Ca^2+^]_*i*_ mobilization and phospholipase C*γ*2 (PLC*γ*2) activation in collagen-activated platelets. Washed platelets were preincubated with 50 and 80 *μ*M ellagic acid or 0.5% DMSO, followed by the addition of 1 *μ*g/mL collagen to trigger (a) relative [Ca^2+^]_*i*_ mobilization and (b) PLC*γ*2 phosphorylation. The data are presented as the means ± S.E.M. (*n* = 4). **P < 0.01 and ***P < 0.001, compared with the control (resting) group; ^#^ *P* < 0.05 and ^###^ *P* < 0.001, compared with the collagen-treated group.

**Figure 3:**
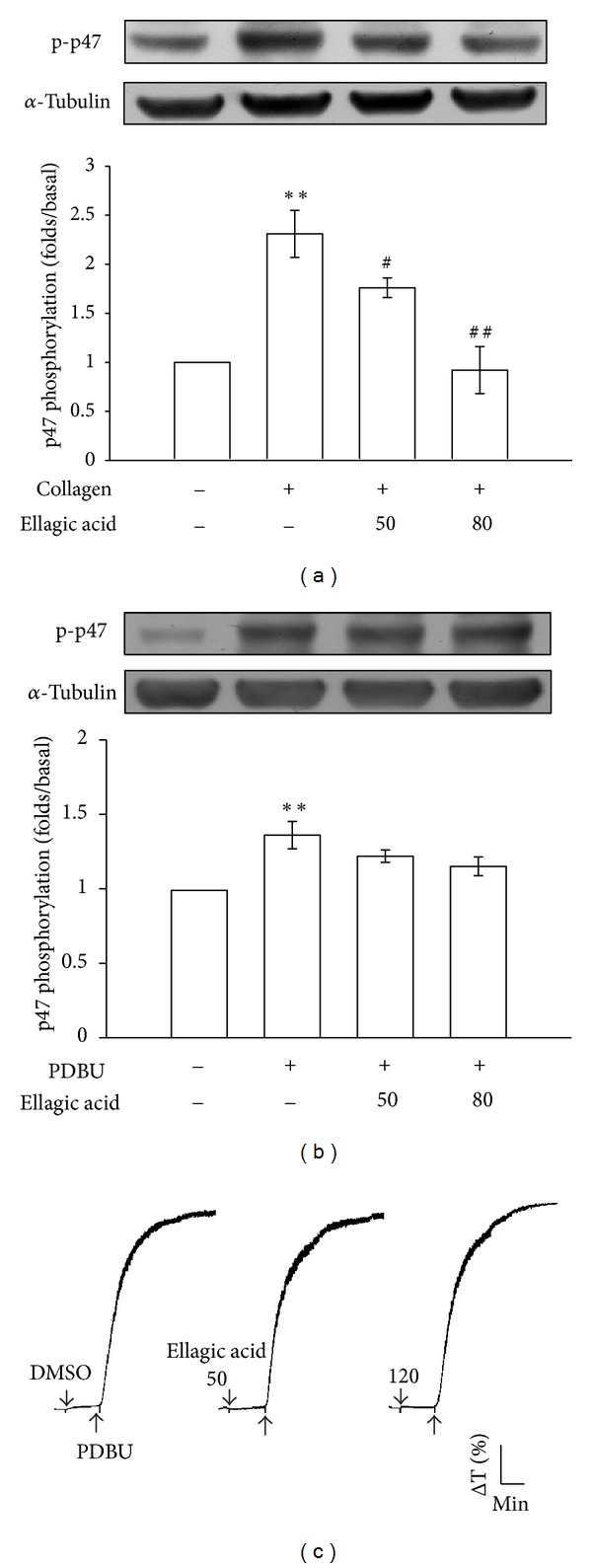
Influence of ellagic acid on protein kinase C (PKC) activation and platelet aggregation in activated platelets. Washed platelets were preincubated with 50 and 80 *μ*M ellagic acid or 0.5% DMSO, followed by the addition of 1 *μ*g/mL collagen or 150 nM PDBu to trigger ((a)-(b)) PKC activation (p47 phosphorylation) and (c) platelet aggregation, as described in the Materials and Methods. The data are presented as the means ± S.E.M. (*n* = 4). **P < 0.01, compared with the control (resting) group; ^#^ *P* < 0.05 and ^##^ *P* < 0.01, compared with the collagen-treated group. The profiles (c) show representative examples of 3 similar experiments.

As shown in Figure 2(a), treatment with 1 *μ*g/mL collagen evoked a marked increase in relative [Ca^2+^]_*i*_ mobilization, and this increase was markedly inhibited in the presence of ellagic acid (resting platelets, 13.3 ± 2.1%; collagen-activated platelets, 73.4 ± 12.3%; with 50 *μ*M ellagic acid, 18.8 ± 3.2%; with 80 *μ*M ellagic acid, 12.4 ± 1.8%; *n* = 4). Furthermore, PLC hydrolyzes phosphatidylinositol 4,5-bisphosphate (PIP_2_) to generate 2 secondary messengers: inositol 1,4,5-trisphosphate (IP_3_) and diacylglycerol (DAG) [15]. DAG activates PKC, inducing protein (p47) phosphorylation and ATP release. Treatment with 50 and 80 *μ*M ellagic acid significantly abolished the phosphorylation of PLC*γ*2 stimulated by collagen (1 *μ*g/mL) (Figure 2(b)). Stimulation of platelets with various agonists induced PKC activation, which then phosphorylated p47 proteins (pleckstrin) [16]. When 1 *μ*g/mL collagen (Figure 3(a)) or 150 nM PDBu (Figure 3(b)) was added to the human platelets, a protein with an apparentmolecular weight of 47 kDa (p47) was predominately phosphorylated, compared with resting platelets. Ellagic acid (50 and 80 *μ*M) markedly inhibited p47 phosphorylation stimulated by collagen, but not by PDBu (Figures 3(a) and 3(b)). In addition, ellagic acid (50 or 80 *μ*M) showed no effects on platelet aggregation stimulated by PDBu (150 nM; Figure 3(c)).

### 3.3. Regulation of MAPK and Akt Phosphorylation by Ellagic Acid

**Figure 4:**
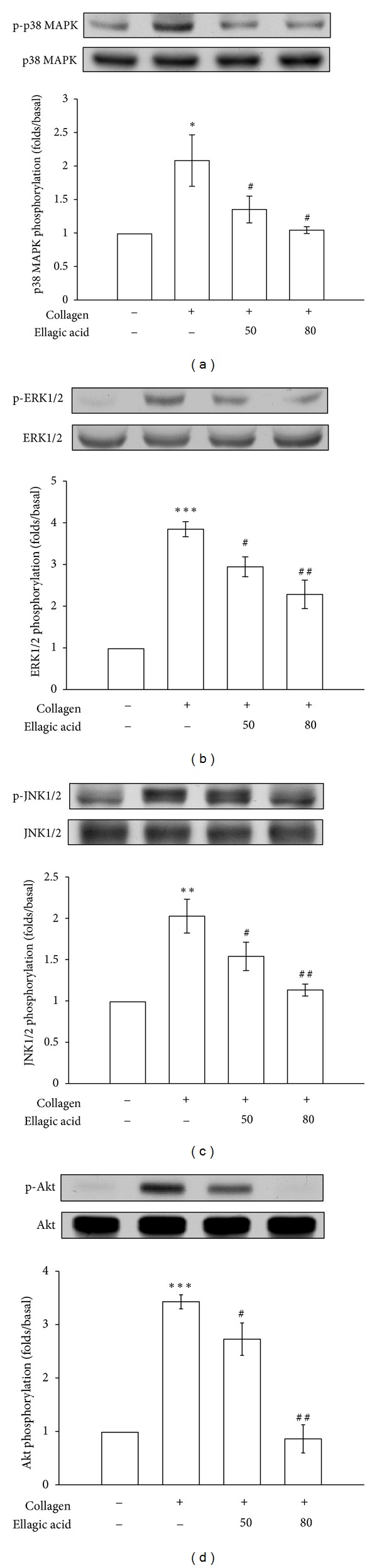
Inhibitory effects of ellagic acid on p38 MAPK, ERK1/2, JNK1/2, and Akt phosphorylation in collagen-activated platelets. Washed platelets (1.2 × 10^9^ cells/mL) were preincubated with 50 and 80 *μ*M ellagic acid or 0.5% DMSO, followed by the addition of 1 *μ*g/mL collagen to trigger platelet activation. The cells were collected, and subcellular extracts were analyzed for (a) p38 MAPK, (b) ERK1/2, (c) JNK1/2, and (d) Akt phosphorylation. The data are presented as the means ± S.E.M. (*n* = 4). *P < 0.05, **P < 0.01, and ***P < 0.001, compared with the control (resting) group; ^#^ *P* < 0.05 and ^##^ *P* < 0.01, compared with the collagen-treated group.

To investigate the possible mechanisms by which ellagic acid inhibits platelet activation, we investigated signaling molecules such as Akt and MAPKs, including p38 MAPK, ERK1/2, and JNK1/2. Ellagic acid (50 or 80 *μ*M) concentration dependently inhibited the phosphorylation of p38 MAPK (Figure 4(a)), ERK1/2 (Figure 4(b)), JNK1/2 (Figure 4(c)), and Akt (Figure 4(d)) stimulated by collagen (1 *μ*g/mL). These results provided evidence of the pivotal role of MAPKs and Akt signals in ellagic acid-mediated inhibition of platelet activation.

### 3.4. Regulatory Role of Ellagic Acid in ROS-Mediated OH^●^ Formation and in p38 MAPK and AKT Activation

**Figure 5:**
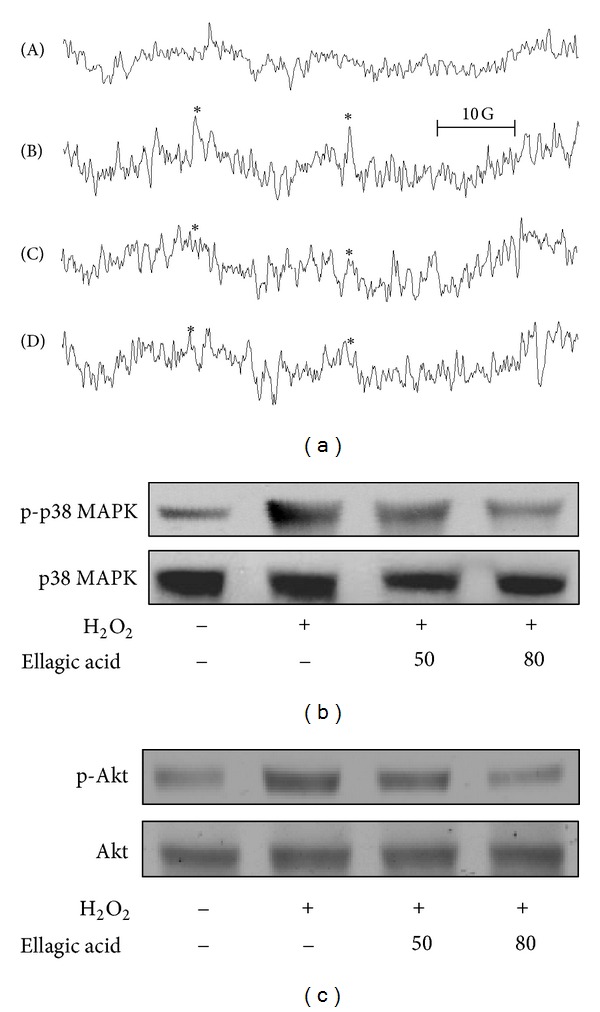
The effects of ellagic acid in regulating hydroxyl radical (OH^●^) formation and p38 MAPK and Akt activation in platelets. (a) For the electron spin resonance (ESR) study, washed platelets were (A) incubated with 0.5% DMSO only (resting group) or preincubated with (B) 0.5% DMSO, (C) 50 *μ*M ellagic acid, and (D) 80 *μ*M ellagic acid, followed by the addition of 1 *μ*g/mL collagen to trigger OH^●^ formation. For other experiments, washed platelets were preincubated with 50 and 80 *μ*M ellagic acid or 0.5% DMSO, followed by the addition of 1 mM hydrogen peroxide to trigger platelet activation. The cells were collected, and subcellular extracts were analyzed for (b) p38 MAPK and (c) Akt phosphorylation. All profiles are representative examples of 4 similar experiments.

A typical ESR signal of hydroxyl radical (OH^●^) formation was triggered in collagen- (1 *μ*g/mL) activated platelets, compared with resting platelets (shown in Figure 5(a) (A, B)). The application of ellagic acid (50 or 80 *μ*M) concentration dependently reduced hydroxyl radical formation stimulated by collagen (Figure 5(a), (C, D)). In addition, ellagic acid (50 or 80 *μ*M) concentration dependently inhibited the phosphorylation of p38 MAPK (Figure 5(b)) and Akt (Figure 5(c)) stimulated by hydrogen peroxide (1 mM).

## 4. Discussion

**Figure 6:**
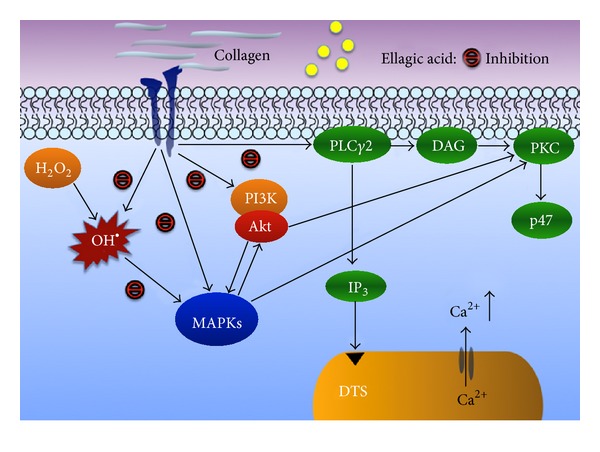
Hypothesis of the inhibitory signaling of ellagic acid in platelet activation. Collagen activates the phospholipase C*γ*2 (PLC*γ*2), diacylglycerol (DAG), protein kinase C (PKC) cascade, and/or hydrogen peroxide (H_2_O_2_) and hydroxyl radical (OH^●^) formation, which then activates PI3-kinase (PI3K)/Akt and mitogen-activated protein kinase (MAPK) activation. Ellagic acid may inhibit both PLC*γ*2-DAG-PKC cascade and hydroxyl radical formation and ultimately inhibits platelet activation. DTS: dense tubular system; IP_3_: inositol 1,4,5-trisphosphate.

Aviram et al. [17] examined the effect of pomegranate juice consumption in atherosclerotic patients who had carotid artery stenosis for 3 years. They found that consumption of pomegranate juice significantly reduced these patients' carotid intima-media thickness (IMT), systolic blood pressure, and LDL oxidation. In the present study, we further showed that ellagic acid effectively prevents platelet activation. Stimulation of platelets by agonists (e.g., collagen) causes marked alterations in phospholipid metabolism. Activation of PLC results in the production of IP_3_ and DAG, which activates PKC, inducing protein phosphorylation (p47) (Figure 6) [18]. PKC activation represents a strategy adopted by cells to allow selected responses to specific activating signals in distinct cellular compartments [19]. The PLC enzymes can be classified into 6 main families, which include at least 13 PLC isoforms: PLC*β* (1 to 4), PLC*γ* (1 and 2), PLC*δ* (1, 3, and 4), PLC*ε* (1), PLC*ζ* (1), and PLC*η* (1 and 2) [20]. PLC*γ*2 is involved in collagen-dependent signaling in platelets [19]. This study showed that both PLC*γ*2 phosphorylation and PKC activity were inhibited by ellagic acid, suggesting that ellagic acid-mediated antiplatelet activity might involve the inhibition of PLC*γ*2-PKC signal pathways (Figure 6). Ellagic acid showed no direct effect on PKC activation because it did not inhibit PDBu-induced PKC activation and platelet aggregation.

The MAPKs consist of 3 major subgroups. The ERKs (p44 ERK1 and p42 ERK2) are involved in cell proliferation, adhesion, and progression [21]. The p38 MAPK and JNKs, which include 46-kDa JNK1 and 55-kDa JNK2 isoforms, appear to be involved in apoptosis [22]. Previous studies have identified ERK1/2, JNK1/2, and p38 MAPK in platelets [22]. The physiopathological roles of JNK1/2 and ERK1/2 remain unclear, but they might be suppressors of *α*_IIb_ *β*_3_ integrin activation or negative regulators of platelet activation [23]. p38 MAPK provides a crucial signal for aggregation caused by collagen. Among the numerous downstream targets of p38 MAPK, the most physiologically relevant in platelets is cytosolic phospholipase A_2_ (cPLA_2_), which catalyzes AA release to produce thromboxane A_2_ (TxA_2_) [24]. Thus, MAPKs (especially for p38 MAPK) appear to have a pivotal role in platelet activation. Moreover, Akt is known to function as one of several downstream effectors of PI3-kinase (Figure 6) [25]. Our previous study showed that both MAPK (i.e., p38 MAPK) and PI3-kinase/Akt are mutually activated as upstream regulators of PKC in activated platelets (Figure 6) [26]. Our studies showed that both MAPKs and Akt activation were inhibited by ellagic acid, suggesting that ellagic acid-mediated antiplatelet activity might also involve the inhibition of MAPKs and/or Akt signal pathways (Figure 6). 

Reactive oxygen species (ROS), including hydrogen peroxide and hydroxyl radicals, are produced during platelet activation and might amplify platelet reactivity during in vivo thrombus formation. Free radical species act as secondary messengers that increase cytosolic Ca^2+^ during the initial phase of platelet activation processes, and PKC is involved in receptor-mediated free radical production in platelets [27]. Studies have shown that some of the hydrogen peroxide produced by platelets is converted into hydroxyl radicals; these findings were based on platelet activation being inhibited by hydroxyl radical scavengers (Figure 6) [27]. Several studies have shown that ROS can trigger MAPKs and Akt phosphorylation in platelets and smooth muscle cells [28, 29]. Moreover, ellagic acid exhibited potent antioxidative activity in scavenging free radical ions in a study that used an indirect method to assay oxygen radical antioxidant capacity [2]. We obtained a similar result from the ESR study, in which ellagic acid was shown to scavenge OH^●^ formation. This finding provided direct evidence of the free radical scavenging activity of ellagic acid. In addition, ellagic acid abolished p38 MAPK and Akt phosphorylation that was stimulated by hydrogen peroxide. Thus, ellagic acid effectively prevented platelet activation, which might be involved (at least in part) in inhibiting the formation of free radicals (Figure 6).

The findings of this study suggest that ellagic acid plays a novel role in antiplatelet activation and can likely be used as a nutritional supplement for prophylactic purposes. Generally, a nutritional or dietary supplement is required to demonstrate a prophylactic effect in humans; however, the response varies across people; hence, it may be impossible to delineate a selection of doses for time-course treatment. This study describes the mechanisms of ellagic acid, administered at the studied doses, in blocking specific signaling events during agonist-induced platelet activation.

In conclusion, this study was the first to show that the antiplatelet activity of ellagic acid might involve an initial inhibition of PLC*γ*2-PKC cascade and hydroxyl radical formation, followed by diminished phosphorylation of MAPKs and Akt. These alterations reduce the relative [Ca^2+^]*i* mobilization and ultimately inhibit platelet aggregation. Ellagic acid was originally considered a chemopreventive agent; however, our study suggests that it might also offer therapeutic potential in the treatment or prevention of thromboembolic disorders.
